# Effects of *Lactobacillus farciminis* and *Lactobacillus rhamnosus* on the duodenal development of specific-pathogen-free broiler chickens

**DOI:** 10.14202/vetworld.2024.2517-2526

**Published:** 2024-11-13

**Authors:** Sabine Eglite, Sintija Jonova, Dace Gorbačevska, Maksims Zolovs, Aija Ilgaza

**Affiliations:** 1 Preclinical Institute, Faculty of Veterinary Medicine, Latvia University of Life Sciences and Technologies, Jelgava, Latvia; 2 Statistics Unit, Riga Stradins University, Riga, Latvia; 3 Department of Biosystematics, Institute of Life Sciences and Technology, Daugavpils University, Daugavpils, Latvia

**Keywords:** duodenum, proliferating cell nuclear antigen, poultry, Ross 308

## Abstract

**Background and Aim:**

The positive effects of *Lactobacillus farciminis* and *Lactobacillus rhamnosus* on growth and feed consumption indicators have been described; however, the underlying mechanisms remain unclear. This study aimed to determine whether the addition of *L. farciminis* CNCM-I-3699 (2.10^10^ GU/g) and *L. rhamnosus* CNCM-I-3698 (2.10^10^ GU/g) to the feed of Ross 308 specific-pathogen-free (SPF) broiler chickens (at a dose of 4 g/10 kg feed) affects live weight gain, the feed conversion ratio (FCR), and duodenal development in SPF broiler chickens.

**Materials and Methods:**

In total, 780 SPF broiler chicks were randomly divided into two groups (three replicates per group) immediately after hatching: The control group (n = 390) and the probiotic group (n = 390). Live body weight (g) and FCR were measured on days 1, 7, 14, 21, 28, and 35 of the study. Histological examinations (hematoxylin and eosin staining) of the duodenum were performed, and the villus height (VH), villus width, crypt depth (CD), muscle layer thickness, and VH: CD ratio were measured. In addition, immunohistochemical examinations were performed to determine the number of proliferating cell nuclear antigen (PCNA)-positive cells.

**Results:**

Feeding a probiotic mixture containing *L. farciminis* and *L. rhamnosus* to SPF broiler chickens for 35 days increased the duodenal absorption area and muscle layer thickness. In addition, it accelerated the histological development of the duodenum, as evidenced by the significantly higher number of PCNA-positive cells within the crypts. Although SPF broiler chickens in the ProL group exhibited greater live weight gain and lower FCR throughout the study, these differences were not statistically significant.

**Conclusion:**

These results suggest that *L. farciminis* and *L. rhamnosus* can serve as additives to SPF broiler chicken feed to promote growth and development.

## Introduction

The quantitative and qualitative composition of the intestinal microbiota in broiler chickens can be altered by stressors in industrial poultry farming. A study by Patterson and Burkholder [1] has shown that stress can decrease the abundance of *Lactobacillus*, disrupt the microbiota, and adversely affect the health and productivity of birds. Since 2006, the use of antibiotics to combat these issues has been prohibited in the European Union [2], necessitating the search for safe alternatives that benefit both animal and human health. One potential strategy to mitigate the harmful effects of stress involves regularly restoring *Lactobacillus* levels through the use of feed additives.

Centuries of human experience and intuitive conclusions regarding the beneficial effects of fermented foods containing lactic acid bacteria on digestive health have been validated by in-depth research. Similarly, previous studies [1, 3] have examined feed additives containing *Lactobacillus* in domestic animals. These bacteria are widely distributed in the environment and constitute a physiologically normal part of the gastrointestinal microbiota in humans and animals, including birds [1, 3]. Research indicates that various strains of the genus *Lactobacillus* typically benefit the host’s immune system, demonstrating high antimicrobial activity against various pathogens, notable adhesion capacity, and resistance to gastric acidity and bile [4, 5]. These characteristics enable a person to overcome the body’s natural protective barriers and survive transit through the digestive tract [6]. Studies involving the feeding of *Lactobacillus rhamnosus* have shown that this probiotic can release antimicrobial substances [7]. It also reduces the transport and toxicity of aflatoxin B1, a toxin harmful to birds [8]. *L. rhamnosus* is recommended to alleviate gastrointestinal disorders, including diarrhea associated with infection, stress, and antibiotic therapy [9]. In addition, *L. rhamnosus*CF (Chen Fu) has been shown to improve several production indicators, such as chicken growth performance, meat quality, and ammonia emission, which are key factors in reducing greenhouse gas emissions [6]. Therefore, *L. rhamnosus* is considered an effective and safe probiotic strain for poultry [10]. Several studies have revealed the positive effects of *Lactobacillus farciminis* on intestinal health and stress resistance [11], overall health status, and live weight gain [12, 13] in animals. It has also been found to mitigate ammonia emissions [6]. Although *L. farciminis* was registered as a feed additive in 2012 [14], as of 2023, expert recommendations stress the need for further research to assess its suitability as a probiotic when fed to broiler chickens [13]. Combining two lactobacilli often enhances the observed probiotic effects. In a study where broiler chicks were fed a combination of *L. rhamnosus* and *L. farciminis* at different doses, the researchers noted a significant improvement in feed digestibility, characterized by a significant decrease in feed intake and feed conversion ratio (FCR), accompanied by an increase in live weight, compared with the unsupplemented groups. However, the underlying reasons for these effects remain unclear [15].

This study aimed to determine whether the simultaneous addition of *L. farciminis* CNCM-I-3699 (2.10^10^ GU/g) and *L. rhamnosus* CNCM-I-3698 (2.10^10^ GU/g) to SPF broiler chicken feed enhances the known effects of these probiotics. Specifically, we investigated the effects of these factors on growth performance and histological indicators of duodenal development in specific-pathogen-free (SPF) broiler chickens.

## Materials and Methods

### Ethical approval

The chickens were housed, fed, and slaughtered by cervical dislocation method in compliance with the Republic of Latvia Cabinet Regulation No. 98, adopted on February 2, 2010, titled “Welfare Requirements for Keeping and Use of Chicken for Meat Production” [16]. In addition, the procedures followed the guidelines outlined in the Ross 308 manual [17]. The research methodology, sample collection, and investigation methods were thoroughly reviewed and approved by the Research Committee of the Faculty of Veterinary Medicine, Latvia University of Life Sciences and Technologies (protocol No. 2021/1).

### Study period and location

The study was conducted from April to December 2021 at Clinical Research Center, Faculty of Veterinary Medicine, Latvia University of Life Sciences and Technologies, Jelgava, Latvia.

### Experimental design and animal management

In total, 780 unsexed Ross 308 SPF broiler chicks were obtained from a commercial hatchery within 2 h of hatching. Rapid acquisition of the chicks and minimization of their exposure to the environment were crucial for reducing the risk of infection by specific pathogens. To ensure this, strict biosecurity measures were implemented from the outset of the study, including restricted access to the premises; disinfection barriers; use of disposable clothing, gloves, and shoes; and twice-daily visits by personnel during feeding. Throughout the study, SPF broiler chickens were not vaccinated.

Upon arrival at the Clinical Research Center, the acclimatization period was 24 h; afterward the SPF broiler chickens were weighed and randomly divided into two groups: The control group (Con, n = 390 (three replicates of 130) and the probiotic group (ProL, n = 390 (three replicates of 130). At the start of the study, the mean weight of the SPF broiler chickens did not differ significantly between the two groups (Con, 44.64 ± 1.92 g; ProL, 45.40 ± 2.03 g; p > 0.05). SPF broiler chickens from each group were housed in two identical 9-m^3^ rooms (bio-chambers), which provided microclimate control (including regulation of temperature, air and litter humidity, incoming and outgoing air temperature and composition, and light/dark mode) and video surveillance. Each bio-chamber was equipped with a stationary automatic watering system and movable feeding tables, which were adjusted according to the age of the chicks before placement. The floor was covered with cleaned coniferous wood shavings with a moisture content of 13%.

The SPF broiler chickens were raised until 35 days of age. The lighting and temperature regimens were established based on prior research and the Ross 308 breeder guidelines [17, 18]. During the 1^st^ week in both groups of chicks, the ambient temperature in the chambers was maintained at 33°C ± 0.6°C, gradually decreasing as the birds grew and reaching 22°C by the end of the study. On the 1^st^ day of the study, the light/dark regimen was set to 23 h of light and 1 h of darkness (23 h/1 h). From day 2, the duration of darkness was gradually extended, reaching 18 h of light and 6 h of darkness (18 h/6 h) by day 7; this pattern was maintained until day 26. From days 27 to 35 of the study, the dark regimen was gradually reduced again to 20 h of light and 4 h of darkness (20 h/4 h).

### Diet and supplementation of *Lactobacillus* spp.

Both study groups were provided with an identical amount of food on the feeding tables twice daily. The poultry-based diet for both groups was formulated specifically for feeding Ross 308 broiler chickens from 1 to 35 days of age and consisted of three phases: Starter (from day 0 to 10, 2975 kcal metabolizable energy [ME]), Grower (from day 11 to 24, 3075 kcal ME), and Finisher (from day 25 to the end of the study [day 35], 3195 kcal ME). The primary protein sources in the feed were wheat grains, soybeans, and canola. The chemical composition of the feed is detailed in Table-1 [19, 20].

**Table-1:** Analytical composition of the basal diet at each feeding stage.

Components	Starter diet	Grower diet	Finisher diet
Metabolizable energy (kcal)	2975	3075	3195
Protein (%)	22.50	21.50	19.50
Fiber (%)	2.40	2.86	2.83
Fat (%)	4.24	5.20	7.22
Ash (%)	4.32	4.73	3.68
Carbohydrates (%)	46.06	46.71	45.53
Water (%)	12.82	11.08	10.98
Other ingredients (%)	5.46	6.12	8.27
Essential amino acids (%)			
Lysine	1.36	1.20	1.14
Methionine	0.84	0.60	0.85
Minerals (%)			
Ca	0.96	1.00	0.78
Na	0.35	0.16	0.19
P	0.50	0.50	0.50
Vitamins			
A (U/kg)	16,900	14,300	13,000
D_3_ (U/kg)	6,500	5,500	5,000
E (mg/kg)	104,0	88.0	80.0
Micronutrients (mg/kg)			
FeSO_4_	22.1	18.7	17.0
Ca (IO_3_)_2_	1.63	1.38	1.25
CuSO_4_	20.8	17.6	16.0
MnO_2_	156.0	132.0	120.0
ZnO	117.0	99.0	90.0
Na_2_SeO_3_	0.39	0.33	0.30

Ca=Calcium, Na=Sodium, P=Phosphorus, FeSO_4_=Iron (II) sulfate, Ca (IO_3_)_2_=Calcium iodate, CuSO_4_=Copper (II) sulfate, MnO=Manganese dioxide, ZnO=Zinc oxide, Na_2_SeO_3_=Sodium selenite

For the ProL group, a mixture of lactic acid bacteria (STI Biotechnologies, France, batch no. 92024406) was added to the feed at an estimated dose of 4 g/10 kg. This mixture contained a heat-inactivated co-culture of the probiotic strains *L. farciminis* CNCM-I-3699 (2.10^10^ GU/g) and *L. rhamnosus* CNCM-I-3698 (2.10^10^ GU/g), supplemented with functional metabolites and bioactive peptides necessary for maintaining bacterial viability. According to the manufacturers, these lactobacilli were sourced from the environment of fermented milk and following heat treatment, retained their cell wall structure. For product stability, the bacteria were preserved on a cereal-based carrier. Upon delivery, the product was stored in a cool, dry, well-ventilated environment in accordance with the manufacturer’s guidelines for a maximum of 18 months from the date of manufacture.

To confirm that SPF broiler chickens in the ProL group received viable probiotic lactic acid bacteria in their digestive tract, we quantified the number of lactobacilli in the duodenal contents of 15 randomly selected SPF broiler chickens from both the Con and ProL groups. These analyses were conducted at the Department of Molecular Biology and Microbiology, Biotechnology Scientific Laboratory, Latvia University of Life Sciences and Technologies [19, 20].

### Growth performance measurements

The live weight of each bird was measured at the start of the study and then weekly (on study days 7, 14, 21, 28, and 35) using calibrated “Soehnl” scales with an accuracy of ±1 g (Soehnl, Germany). The FCR for each group was calculated using the formulas described by Chen *et al*. [6]. Data recording and calculations were conducted for consecutive study periods: Days 1–7, 8–14, 15–21, 22–28, and 29–35.

### *Lactobacillus* spp. quantity control in duodenal contents

Despite adhering to the manufacturer’s recommendations regarding the storage and feeding of feed supplements containing two species of *Lactobacillus* to SPF broiler chickens, we aimed to confirm that the *Lactobacillus* spp. counts in the digestive tract of birds in the ProL group were higher than those in the Con group. At the end of the study (day 35), after collecting histological and bacteriological samples, a total sample (50 mL) of the duodenal contents from each group was obtained and subsequently frozen at −22°C.

The samples were analyzed at the Latvia University of Life Sciences and Technologies (LBTU) Research Laboratory of Biotechnology Division of Molecular Biology and Microbiology. According to the information provided by the laboratory, the isolation and quantification of *Lactobacillus* spp. were performed using Man-Rogosa-Sharpe (MRS) agar with polysorbate 80 (MRS agar with Tween® 80, Biolife, Italy). From the total sample of the initial duodenal contents, 1 mL of suspension and its decimal dilutions were transferred into parallel sterile Petri dishes. Next, 10 mL of 45°C–47°C MRS agar was poured into the dishes and allowed to solidify. To maintain microaerophilic conditions, the plates were then overlaid with an additional 10 mL–15 mL of MRS agar and incubated at 36°C ± 1°C for 72 ± 2 h.

After incubation, dilution plates on which the number of *Lactobacillus* spp. colonies did not exceed 300 colony-forming units (CFU) were selected and counted. For control purposes, characteristic colonies were confirmed using the Gram staining method and the catalase test because *Lactobacillus* spp. typically present as Gram-positive cocci or rods with a negative catalase test reaction [21]. The number of *Lactobacillus* spp. bacteria in 1 g of the test sample was expressed as log10 CFU/g in accordance with the Latvian National Standard, European Standard, International Organization for Standardization 8199:2019 (LVS EN ISO 8199:2019) standard. Because this analysis was informative in nature, an in-depth identification of *Lactobacilli* species was not performed.

### Duodenal sample collection and examination

To obtain samples for duodenal microscopic examination, we randomly selected and euthanized (SPF broiler chickens were stunned using the cervical dislocation method and bled) 10 chicks from each group on days 1, 7, 14, 21, 28, and 35. Thus, in each repetition, samples were collected from 120 birds (Con, n = 60; ProL, n = 60) for histological examination.

Tissue samples were collected from the middle of the duodenal flexure. Histological examinations were conducted in the duodenal segment nearer to the muscular stomach. However, accurately defining the exact distance from the muscular stomach was challenging because this distance increased with increasing chick growth.

The obtained samples were fixed in a 10% buffered neutral formalin solution. The fixed tissue samples were then cut and placed in tissue cassettes. Subsequent sample preparation for histological examination was conducted in the Laboratory of Comparative Pathology at the Pre-clinical Institute of the Faculty of Veterinary Medicine, LBTU. The samples were dehydrated by removing water and formalin in a series of increasing alcohol concentrations using a LEICA ASP200S tissue processor (Leica, Germany). Following dehydration, xylene was used to remove the alcohol from the tissues. Processed tissues were embedded in paraffin blocks using the Tissue Embedding System TES 99 (Medite, Germany).

### Sample preparation and histological examination

In the Laboratory of Comparative Pathology, paraffin-embedded samples were sectioned into 4-μm-thick slices using a microtome and mounted on slides (Deltalab, Spain) for hematoxylin and eosin staining. The sections were fixed for 16 h in a thermostat at 37°C, followed by deparaffinization with xylene and rehydration through a graded series of alcohols. The samples were then stained using the standard hematoxylin–eosin method described by Carson [22]: Application of Mayer’s hematoxylin (Bio-Optica, Italy) for 2 min, a rinse in running water for 5 min, application of Eosin G Y solution 0.5%, alcoholic (Diapath, Italy) for 3 min, and a final rinse in running water for 3 min.

For histological examination of the duodenum, we selected 10 visible and parallel-oriented crypt/villi units from each cross-section of the intestine. The villi were selected based on the presence of a clearly visible lamina propria. Villus height (VH) was measured from the tip of the villi to the villus/crypt junction, and crypt depth (CD) was determined as the depth of intussusception between adjacent villi. For each intestinal sample, 10 villi were performed in 5 fields of view. The examination was performed using a Leica DM2500 light microscope, and all images were captured using a Leica DFC450 digital camera (Leica, Germany).

### Preparation and examination of immunohistochemical samples

Proliferating cell nuclear antigen (PCNA) immunohistochemical staining was performed at the Creative Bioarray Laboratory (USA) on formalin-fixed, paraffin-embedded duodenal tissue samples. The laboratory provided the following staining protocol:

- Slide preparation: Formalin-fixed, paraffin-embedded slides were prepared at a thickness of 4 μm.
- Antigen retrieval: The specimens were immersed in citrate buffer (10 mM citrate buffer, 0.05% Tween 20, pH 6.0), microwaved for 3 min, and then steamed at 95°C for 15 min.
- Protein blocking: Normal goat serum block for 15 min.
- Primary antibody incubation: 4°C overnight.
- Endogenous peroxidase blocking: 1% hydrogen peroxide (H_2_O_2_) in phosphate-buffered saline for 15 min.
- Secondary antibody incubation: VisUCyte horseradish peroxidase goat antimouse polymer (Bio-Techne, USA) was incubated for 60 min at room temperature.
- 3,3’-Diaminobenzidine (DAB) staining: 25 μL stock DAB was mixed with 1 mL of DAB buffer plus H_2_O_2_ for 1 min.
- Counterstaining: Mayer’s hematoxylin solution for 60 s.

The experimental samples were chicken duodenum: Immunohistochemistry (IHC) 2175, mouse monoclonal (PC10) to PCNA (Cat. # Fab29; Abcam, UK), diluted 1:20,000. The Con samples were human tonsil: Mouse monoclonal (PC10) to PCNA (Cat. # ab29; Abcam), with positive and negative controls.

The samples stained with the PCNA immunohistochemical method were examined using an Axiolab 5 light microscope (Zeiss, Germany) equipped with Labscope Version 3.4.2 (Zeiss), which enables cell counting in a designated area.

PCNA-positive nuclei were counted among 10 clearly visible, uniformly oriented villi in accordance with the protocol described by Bologna-Molina *et al*. [23]. The examination area was marked on the middle third of the villi. Using the Zeiss examination program (Labscope Version 3.4.2), the area was determined and fixed in μm^2^. All visible PCNA-positive nuclei within the marked area were then counted, regardless of staining intensity. PCNA-positive nuclei were also counted in 10 clearly visible, uniformly oriented crypts using the same method. All images were captured using a digital camera Axiocam 208 color (Zeiss).

### Statistical analysis

To compare quantitative variables (weight and histological parameters) between the Con and ProL groups, an independent samples t-test was conducted because the data were normally distributed (assessed by the Shapiro-Wilk test with Q-Q plot inspection) and homogeneous (assessed by Levene’s test). The Mann-Whitney U test was used to compare the number of PCNA-positive cells between the Con and ProL groups.

To compare the above-mentioned parameters between different time periods, a one-way repeated-measures analysis of variance or Friedman’s test was applied as appropriate. Spearman’s test was used to assess the correlation between histological parameters (VH, villus width, CD, and muscle layer thickness) and the number of PCNA-positive cells. Statistical analyses were conducted using Jamovi software (v2.3.28) [24]. The results are presented as mean ± standard deviation unless otherwise stated. Differences were considered statistically significant at p < 0.05.

## Results

### Growth rates

The weights of the birds at the beginning of the study were not significantly different between the two groups (p > 0.05), indicating that subsequent results were not affected (Table-2). The mean live weight and FCR of birds at all age stages were also not significantly different between the two groups (p > 0.05).

**Table-2:** Growth rate of specific-pathogen-free broiler chickens.

Parameter	Day	Mean (95% CI)		p-value

		Control group	Probiotic group	
Live weight (g)	0	43.8 (42.1–45.5)	44.5 (42.5–46.4)	0.945
	7	229.2 (225.6–232.8)	216.7 (216.4–223.1)	0.831
	14	582.2 (572.8–591.5)	588.6 (578.9–598.1)	0.834
	21	1154.1 (1136.7–1171.5)	1166.2 (1147.4–1184.9)	0.979
	28	1957.7 (1931.0–1984.4)	1961.9 (1935.0–1988.9)	0.989
	35	2828.0 (2784.2–2871.8)	2835.7 (2791.7–2879.7)	0.947
Feed conversion ratio	1–7	1.0 (0.8–1.3)	1.0 (0.8–1.2)	0.998
	8–14	1.1 (0.9–1.3)	1.1 (0.9–1.3)	0.794
	15–21	1.1 (0.9–1.3)	1.1 (0.9–1.3)	0.835
	22–28	1.2 (1.1–1.3)	1.2 (1.1–1.3)	0.944
	29–35	1.3 (1.2–1.4)	1.3 (1.2–1.4)	0.757

CI=Confidence interval

### Lactobacilli content in the duodenum

The mean number of lactobacilli in the duodenal contents, as assessed in three replicates, was 5.41 × 10^6^ in the ProL group and 4.64 × 10^6^ in the Con group. The higher number of lactobacilli in the duodenal contents of the ProL group suggests that these birds received additional *L. farciminis* and *L. rhamnosus* as feed (these bacteria are capable of growing and reproducing in the digestive tract).

### Histological examinations

VH was significantly greater in the ProL group on day 7 (p < 0.05); however, the effect size indicated a low practical significance of this difference (Table 3). A significantly greater VH in the ProL group was also observed on days 21, 28, and 35 (p < 0.01), and the effect size indicated the average practical meaning of this difference. Faster villus growth was observed from days 1 to 14 and from day 21 to the end of the study in both the ProL and Con groups (p < 0.01).

**Table-3:** Duodenum histological examinations.

Parameter (μm)	Day	Control M (SD)	Probiotic M (SD)	p-value	d
Villi length	1 d	532 (127)^a^	528 (116)^a^	0.721	NA
	7 d	1142 (175)^b^	1174 (168)^b^	0.024	0.18
	14 d	1523 (241)^c^	1551 (206)^c^	0.135	NA
	21 d	1509 (206)^c^	1583 (172)^c^	<0.001	0.39
	28 d	1580 (222)^d^	1697 (254)^d^	<0.001	0.49
	35 d	1686 (207)^e^	1758 (204)^e^	<0.001	0.35
p-value		<0.001	<0.001		
Villi width	1 d	89 (20)^a^	88 (19)^a^	0.752	NA
	7 d	166 (37)^b^	173 (37)^b^	0.025	0.18
	14 d	200 (42)^c^	214 (45)^c^	<0.001	0.30
	21 d	215 (46)^d^	213 (42)^c^	0.642	NA
	28 d	229 (41)^e^	240 (50)^d^	0.002	0.25
	35 d	246 (49)^f^	246 (48)^d^	0.888	NA
p-value		< 0.001	< 0.001		
Crypt depth	1 d	74 (12)^a^	76 (13)^a^	0.152	NA
	7 d	143 (31)^b^	148 (22)^b^	0.031	0.18
	14 d	190 (29)^c^	185 (32)^c^	0.078	NA
	21 d	199 (27)^d^	206 (31)^d^	0.003	0.25
	28 d	214 (34)^e^	220 (33)^e^	0.021	0.19
	35 d	225 (39)^e^	239 (43)^f^	<0.001	0.33
p-value		< 0.001	< 0.001		
Muscle layer thickness	1 d	70 (20)^a^	76 (20)^a^	<0.001	0.31
	7 d	116 (35)^b^	120 (30)^b^	0.187	NA
	14 d	129 (37)^c^	135 (39)^c^	0.040	0.17
	21 d	142 (36)^d^	142 (33)^d^	0.950	NA
	28 d	157 (37)^e^	163 (41)^e^	0.065	NA
	35 d	170 (55)^f^	174 (47)^f^	0.328	NA
p-value		< 0.001	< 0.001		
Villi length/crypt depth	1 d	7.3 (2.1)^a^	7.1 (1.7)^a^	0.222	NA
	7 d	8.5 (4.3)^b^	8.3 (3.8)^b^	0.516	NA
	14 d	8.2 (1.5)^c^	8.5 (1.4)^c^	0.002	0.26
	21 d	7.7 (1.2)^a^	7.8 (1.2)^d^	0.185	NA
	28 d	7.7 (4.2)^a^	7.8 (1.5)^d^	0.655	NA
	35 d	8.1 (6.2)^d^	8.1 (6.8)^b^	0.901	NA
p-value		< 0.001	< 0.001		

M=Mean, SD=Standard deviation, d=Cohen’s d (effect size). Values with different superscript letters (a–e) in the same column are significantly different (p < 0.05)

The villus width was significantly greater in the ProL group on day 7 (p < 0.05), but the effect size indicated a low practical meaning of this difference. In addition, the villus width in the ProL group was significantly greater on days 14 and 28 (p < 0.01), and the effect size indicated the average practical meaning of this difference. Significant differences in villus width were observed between age groups within both groups (p < 0.01). In the Con group, villus width gradually increased throughout the study period, whereas in the ProL group, a faster increase in villus width was observed from days 7 to 14 and from days 21 to 28.

CD was significantly greater in the ProL group on days 7, 21, 28, and 35. Although the difference was statistically significant on days 7 and 28 (p < 0.05), the effect size indicated a low practical meaning of the difference. On days 21 and 35, however, the effect size indicated the average practical significance of this difference (p < 0.01). Within both the Con and ProL groups, statistically significant differences in CD were observed between age groups (p < 0.01), except in the Con group between days 28 and 35, during which rapid growth of crypts was not detected (p > 0.05).

The muscle layer thickness was significantly greater in the ProL group on day 1 (p < 0.01), with the effect size indicating the average practical meaning of this difference, as well as on day 14 (p < 0.05), with the effect size indicating the low practical meaning of this difference. Within both groups, statistically significant differences in muscle layer thickness were observed between age groups (p < 0.01).

The VH: CD ratio was significantly higher in the ProL group on day 14 (p < 0.05), and the effect size indicated the average practical significance of this difference. In both study groups, VH: CD decreased on days 21 and 28.

VH significantly differed between the Con and ProL groups (p < 0.01). In addition, significant differences were found in villus width, CD, and muscle layer thickness (p < 0.05); the exception was the VH:CD, which showed no significant differences (p > 0.05). Although the differences in the specific indicators were statistically significant, the effect size indicated that these differences had a low practical meaning (Table-4).

**Table-4:** Overall mean scores for histological examination of the duodenum.

Parameter (μm)	Control group		Probiotic group		p-value	d
	
	M	SD	M	SD		
Villi length	1332.2	436.9	1383.9	462.3	< 0.001	0.12
Villi width	191.2	65.1	195.9	67.3	0.034	0.10
Crypt depth	174.6	58.9	179.2	61.8	0.023	0.10
Muscle layer thickness	130.8	49.4	135.1	47.9	0.009	0.10
Villi length/crypt depth ratio	7.9	3.7	7.9	3.4	0.831	NA

M=Mean, SD=Standard deviation, d=Cohen’s d (effect size)

### IHC examinations

The number of PCNA-positive cells in crypts and villi is expressed per 1 mm^2^. At all age stages, the number of PCNA-positive cells was significantly higher in the ProL group than in the Con group (p < 0.01).

No differences were observed between the groups in the number of PCNA-positive villi cells on day 7. However, on days 21 and 35, the number of positive cells was significantly higher in the Con group (p < 0.01 and p < 0.05, respectively) (Table-5).

**Table-5:** Number of PCNA-positive duodenal cells.

Parameter	Age	Crypts			Villi		
	
		Control group	Probiotic group	p-value	Control group	Probiotic group	p-value
Cells/mm^2^	Days	M (Q1–Q3)	M (Q1–Q3)		M (Q1–Q3)	M (Q1–Q3)	
	7	8.5 (7.3–9.7)^a^	9.6 (8.6–11.8)^a^	< 0.001	2.2 (1.9–3.0)^a^	2.3 (1.9–2.6)^a^	0.258
	21	6.8 (5.6–7.3)^b^	6.9 (5.4–9.2)^b^	< 0.001	1.4 (1.2–1.5)^b^	0.8 (0.6–1.0)^b^	< 0.001
	35	4.4 (3.7–5.1)^c^	4.6 (3.5–6.0)^c^	< 0.001	0.9 (0.7–1.1)^c^	0.7 (0.6–0.8)^b^	0.021
		p < 0.001	p < 0.001		p < 0.001	p < 0.001	

Values with different superscript letters (a–c) in the same column are significantly different (p < 0.05). M=Median, Q1 and Q3=The first and third quartiles, PNCA=Proliferating cell nuclear antigen

The results showed that the number of PCNA-positive cells decreased with age. Significant differences in the numbers of cells were observed between days 7, 21, and 35 in both the crypts and villi within each group (p < 0.01); however, no significant differences were observed in the villi of the ProL group between days 21 and 35 (p > 0.05). No significant correlation was observed between the number of PCNA-positive cells in crypts and villi (p > 0.05) (Table-5).

### Correlation

A statistically significant negative correlation was found between histological parameters (VH, villus width, CD, and muscle layer thickness) and the number of PCNA-positive cells. As the birds grew, the histological indicators also increased, including VH (r = −0.254), villus width (r = −0.184), CD (r = −0.329), and muscle layer thickness (r = −0.268), whereas the number of PCNA-positive cells decreased (p < 0.01).

## Discussion

Consistent with findings from other studies [25–28], our study did not observe significant differences in growth indicators (p > 0.05), although the ProL group consistently showed numerically higher live weights throughout the study period and at its conclusion (Table-2). Our study provided optimal housing conditions and specific-pathogen-free environments, which contributed to good live weight gain in both groups. Consequently, the high live weight gain was not significantly different between the ProL and Con groups.

In real-world production settings, achieving such ideal circumstances is challenging, and we assumed that the impact of ProLs would be more pronounced under less optimal conditions. However, some studies have shown the opposite effect. Shah *et al*. [29] reported a significant increase in live weight when broiler chickens were fed ProLs containing *Enterococcus faecium* and *Pediococcus acidilactici*. Similarly, Chen *et al*. [6] observed a substantial increase in live weight when *L. rhamnosus* was fed.

This study also revealed no significant differences in FCR (Table-2). Similar results were obtained by Qorbanpour *et al*. [30], who fed a multistrain ProL containing *L. acidophilus* and *L. casei* to Ross 308 broiler chickens without improving the FCR. A study by Awad *et al*. [4], in which a ProL containing *Lactobacillus* spp. was fed for 35 days and they did not observe a significant improvement in the FCR in the ProL group, although it was numerically lower. Similarly, Such *et al*. [27] found no improvement in feed conversion when *L. farciminis* was added to the feed.

However, in a study where broiler chickens were fed equal concentrations of thermally inactivated *Bacillus subtilis* and *L. acidophilus*, a significant improvement in FCR was achieved [26]. In addition, feeding multistrain probiotics to broiler chickens [31] and feeding *L. rhamnosus* to broiler chickens [6] improved FCR.

Rapid growth and development of the villi were observed from the beginning of the study to day 7, during which the height, width, and depth of the crypts doubled (Table-3). This period of relatively rapid growth continued until day 14, coinciding with the rapid increase in the live weight of the birds. Cao *et al*. [12] reported that birds with more fully developed intestinal morphologies tend to have better growth rates. After day 14, villi growth and development slowed but continued at a more gradual pace. Throughout all age stages, better villus growth was observed in the ProL group. At the end of the study (day 35), the VH and CD were significantly higher in the ProL group than in the non-ProL group. By contrast, Awad *et al*. [4] did not observe significant increases in VH or CD in the duodenum compared with the control group. Similarly, in a study by Such *et al*. [27], *L. farciminis* was added to the feed of Ross 308 broiler chickens at 5 × 10^9^ CFU/kg and observed that the VH and CD in the ileum were lower in the probiotic group than in the control group at the end of the study.

During the study period, the live weight of the birds rapidly increased. The VH: CD ratio decreased from days 14 to 21, indicating slower villi growth during this period (p > 0.05). However, from day 28 to the end of the study, the VH: CD increased again because the villi growth was faster in this age group (Table-3).

The VH: CD ratio is an important indicator of the absorption of nutrients and, consequently, the growth of birds. A higher VH: CD ratio is associated with better absorption of nutrients in the digestive tract [32]. In both the Con and ProL groups, the VH: CD ratio increased between age groups until day 14 (1–7 and 7–14 days of age, p < 0.01). This increase can be attributed to the rapid growth of villi, as evidenced by the gradual increase in CD throughout the study period (p < 0.01). During this period, there was also a rapid increase in the live weight of the birds. Similar results and relationships were obtained by Awad *et al*. [4]. As the birds continued to grow, the VH: CD ratio decreased from days 14 to 21, indicating a slowing of villus growth (p > 0.05). However, from day 28 to the end of the study, the VH: CD ratio increased again, suggesting faster villus growth during this stage, with crypt development remaining at the previous level. There was no significant difference in the VH: CD ratio between the groups at the end of the study. Contrarily, results were reported by Awad *et al*. [4], who found a significantly higher VH: CD ratio in the probiotic-enriched group. In addition, numerically higher VH: CD ratios were reported by Such *et al*. [27], Nguyen *et al*. [32], and Liao *et al*. [33]. These studies also showed a positive correlation between the VH: CD ratio and the presence of lactobacilli in the intestinal tract.

Histological examinations indicated that, on average, villus development was significantly faster and better in the ProL group throughout the entire study (Table-4). Similar results were achieved in studies conducted by Awad *et al*. [4] and Elhassan *et al*. [34] when broiler chickens were fed probiotics.

The impact of feeding *L. farciminis* and *L. rhamnosus* on the growth and division of broiler chickens was evident in the significantly higher number of PCNA-positive cells in the crypts of the ProL group at all age stages (Table-5). A relationship was observed between the number of PCNA cells in the crypts and the CD count; specifically, the ProL group, which had a significantly higher CD count, also showed a higher number of PCNA-positive cells within the crypts. Similar results were reported by Calik *et al*. [35], who observed a greater CD and a higher number of PCNA-positive cells in broiler chickens fed synbiotics. Unexpectedly, no relationship was observed between the number of PCNA-positive cells within the villi and villus parameters (VH and villus width). Although we expected that the number of PCNA-positive cells would be higher in the villi of the group with faster growth, this correlation was not evident in this study, similar to other studies [35, 36]. The ProL group exhibited more radial villi growth, whereas a higher number of PCNA-positive cells were observed in the Con group. As the birds grew, the number of PCNA-positive cells remained significantly higher in the crypts but might have decreased in the villi while maintaining high VH and villus width. This pattern was observed in SPF broiler chickens of the ProL group, suggesting that SPF broiler chickens that received *L. farciminis* and *L. rhamnosus* developed faster, as indicated by the high rates of live weight gain.

As the birds grew, there was an overall increase in histological indicators, such as the length, width, and depth of the crypts, and the thickness of the muscle layer. However, there was a concurrent decrease in the number of PCNA-positive cells (p < 0.01). This negative correlation between the number of PCNA-positive cells and bird growth was also observed by Petrusewicz-Kosinska *et al*. [37], who observed a statistically significant decrease in PCNA-positive cell proliferation in the pineal gland as turkeys grew. Higher PCNA-positive cell counts are more often observed in the early stages of bird growth due to the rapid growth and development of birds at younger ages, especially in fast-growing breeds, as observed in the present study of Ross 308 SPF broiler chickens. The rapid growth and division of cells in birds decrease with growth.

## Conclusion

Feeding a mixture containing *L. farciminis* and *L. rhamnosus* probiotics to SPF broiler chickens for 35 days increased the absorption area of the duodenum. This was evidenced by increased intestinal histological indicators in SPF broiler chickens at different age stages (VH, villus width, CD, VH: CD ratio, and muscle layer thickness) and a significantly higher number of PCNA-positive cells within the crypts. These findings suggest that feeding SPF broiler chickens a mixture of *L. farciminis* and *L. rhamnosus* positively affects live weight gain and feed conversion, indicating potential growth-promoting benefits. However, these changes did not reach statistical significance. In addition to the effects on growth parameters, this study highlights the accelerated histological development of the duodenum in SPF broiler chickens fed *L. farciminis* and *L. rhamnosus*. This suggests that the probiotic mixture may contribute to the overall intestinal health and development of SPF broiler chickens.

## Authors’ Contributions

SE and AI: Conceived, designed, and coordinated the study. SE and AI: Performed the experiment. MZ: Performed the statistical analysis. DG: performed histological examination. SE, AI, and SJ: Analyzed and interpreted the data. SE, AI, SJ, and DG: Participated in the preparation and revision of the manuscript. All authors have read, reviewed, and approved the final manuscript.

## References

[1] Patterson J.A, Burkholder K.M (2003). Application of prebiotics and probiotics in poultry production. Poult. Sci.

[2] Regulation of the European Parliament and Council No.1831/2003 Additives Used in Animal Feeding.

[3] Mao X, Gu C, Hu H, Tang J, Chen D, Yu B, He J, Yu J, Luo J, Tian G (2016). Dietary *Lactobacillus rhamnosus* GG supplementation improves the mucosal barrier function in the intestine of weaned piglets challenged by porcine rotavirus. PLoS One.

[4] Awad W.A, Ghareeb K, Abdel-Raheem S, Böhm J (2009). Effects of dietary inclusion of probiotic and synbiotic on growth performance, organ weights, and intestinal histomorphology of broiler chickens. Poult. Sci.

[5] Sugiharto S, Ranjitkar S (2019). Recent advances in fermented feeds towards improved broiler chicken performance, gastrointestinal tract microecology and immune responses:A review. Anim. Nutr.

[6] Chen F, Gao S.S, Zhu L.Q, Qin S.Y, Qiu H.L (2018). Effects of dietary *Lactobacillus rhamnosus* CF supplementation on growth, meat quality, and microenvironment in specific pathogen-free chickens. Poult. Sci.

[7] Toki S, Kagaya S, Shinohara M, Wakiguchi H, Matsumoto T, Takahata Y, Morimatsu F, Saito H, Matsumoto K (2008). *Lactobacillus rhamnosus* GG and *Lactobacillus casei* suppress *Escherichia coli*-induced chemokine expression in intestinal epithelial cells. Int. Arch. Allergy Immunol.

[8] Gratz S, Wu Q.K, El-Nezami H, Juvonen R.O, Mykkänen H, Turner P.C (2007). *Lactobacillus rhamnosus* strain GG reduces aflatoxin B1 transport, metabolism, and toxicity in Caco-2 cells. Appl. Environ. Microbiol.

[9] Reid G, Jass J, Sebulsky M.T, McCormick J.K (2003). Potential uses of probiotics in clinical practice. Clin. Microbiol. Rev.

[10] Commission Implementing Regulation (EU) 2017/1903, Concerning the Authorisation of the Preparations of Pediococcus parvulus DSM 28875, *Lactobacillus casei* DSM 28872 and *Lactobacillus rhamnosus* DSM 29226 as Feed Additives for All Animal Species.

[11] Ait-Belgnaoui A, Han W, Lamine F, Eutamene H, Fioramonti J, Bueno L, Theodorou V (2006). *Lactobacillus farciminis* treatment suppresses stress induced visceral hypersensitivity:A possible action through interaction with epithelial cell cytoskeleton contraction. Gut.

[12] Cao G.T, Zeng X.F, Chen A.G, Zhou L, Zhang L, Xiao Y.P, Yang C.M (2013). Effects of a probiotic, *Enterococcus faecium*, on growth performance, intestinal morphology, immune response, and cecal microflora in broiler chickens challenged with *Escherichia coli* K88. Poult. Sci.

[13] Azimonti G, Bastos M.L, Christensen H, Dusemund B, Kouba M, Kos Durjava M, López-Alonso M, López Puente S, Marcon F, Mayo B, Pechová A, Petkova M, Ramos F, Sanz Y, Villa R.E, Woutersen R, Cocconcelli P.S, Glandorf B, Herman L, Prieto Maradona M, Saarela M, Anguita M, Pettenati E, Brozzi R (2020). Safety and efficacy of Biacton®(*Lactobacillus farciminis* CNCM I-3740) as a feed additive for chickens for fattening, turkeys for fattening and laying hens. EFSA J.

[14] Commission Implementing Regulation (EU) No1018/2012, Regulation as Regards the Maximum Content of Certain Micro-organisms in Complete Feeding Stuffs.

[15] Bampidis V, Azimonti G, Bastos M.L, Christensen H, Dusemund B, Kos Durjava M, Kouba M, López-Alonso M, López Puente S, Marcon F, Mayo B, Pechová A, Petkova M, Ramos F, Sanz Y, Villa R.E, Woutersen R, Cocconcelli P.S, Glandorf B, Prieto Maradona M, Saarela M, Rychen G, Pettenati E, Brozzi R (2020). Safety and efficacy of Sorbiflore®ADVANCE (*Lactobacillus rhamnosus* CNCM I-3698 and *Lactobacillus farciminis* CNCM I-3699) as a feed additive for chickens for fattening. EFSA J.

[16] Republic of Latvia Cabinet Regulation No.98, Welfare Requirements for Keeping and Use of Chickens for Meat Production.

[17] Broiler Management Manual ROSS.

[18] Cassuce D.C, Tinôco I.F.F, Baêta F.C, Zolnier S, Cecon P.R, Vieira M.F.A (2012). Thermal comfort temperature update for broiler chickens up to 21 days of age. Eng. Agric.

[19] Eglite S, Mancevica L, Ilgaza A (2023). Effects of dietary supplementation of *Lactobacillus farciminis* and *Lactobacillus rhamnosus* on growth and production indicators of broiler chickens. J. Worlds Poult. Res.

[20] Eglite S, Ilgaza A, Mancevica L, Zolovs M (2023). The effects of *Lactobacillus farciminis* and *Lactobacillus rhamnosus* on growth, blood biochemical, and meat quality indicators of specific pathogen-free broiler chickens. Vet. Med. Int.

[21] Mokoena M.P (2017). Lactic acid bacteria and their bacteriocins:Classification, biosynthesis and applications against uropathogens:A mini-review. Molecules.

[22] Carson F.L (1997). Histotechnology:A Self-Instructional Text.

[23] Bologna-Molina R, Damián-Matsumura P, Molina-Frechero N (2011). An easy cell counting method for immunohistochemistry that does not use an image analysis program. Histopathology.

[24] The Jamovi Project, Jamovi (Version 2.3) (Computer Software).

[25] Sugiharto S, Isroli I, Yudiarti T, Widiastuti E (2018). The effect of supplementation of multistrain probiotic preparation in combination with vitamins and minerals to the basal diet on the growth performance, carcass traits, and physiological response of broilers. Vet. World.

[26] Zhu C, Gong L, Huang K, Li F, Tong D, Zhang H (2020). Effect of heat-inactivated compound probiotics on growth performance, plasma biochemical indices, and cecal microbiome in yellow-feathered broilers. Front. Microbiol.

[27] Such N, Molnár A, Farkas V, Pál L, Husvéth F, Koltay I, Rawash M, Mezőlaki A, Dublecz K (2020). Feeding two single strain probiotic bacteria and wheat bran failed to modify the production traits but altered some gut characteristics in broiler chickens. J. Cent. Eur. Agric.

[28] Astuti F.D, Sugiharto S, Yudiarti T, Widiastuti E, Wahyuni H.I, Ayaşan T (2022). Growth performance, blood variables, intestinal bacterial content, and morphological measurements of broilers supplemented with *Lactobacillus*
*casei*-fermented mixture of red rice and aromatic ginger. Vet. World.

[29] Shah S.M.T, Islam M.T, Zabin R, Roy P.C, Meghla N.S, Jahid I.K (2021). Assessment of novel probiotic strains on growth, hematobiochemical parameters, and production costs of commercial broilers in Bangladesh. Vet. World.

[30] Qorbanpour M, Fahim T, Javandel F, Nosrati M, Paz E, Seidavi A, Ragni M, Laudadio V, Tufarelli V (2018). Effect of dietary ginger (*Zingiber officinale* roscoe) and multi-strain probiotic on growth and carcass traits, blood biochemistry, immune responses and intestinal microflora in broiler chickens. Animals (Basel).

[31] Hussein E, Selim S (2018). Efficacy of yeast and multi-strain probiotic alone or in combination on growth performance, carcass traits, blood biochemical constituents, and meat quality of broiler chickens. Livest. Sci.

[32] Nguyen D.T.N, Le N.H, Pham VV, Eva P, Alberto F, Le H.T (2021). Relationship between the ratio of villous height:Crypt depth and gut bacteria counts as well production parameters in broiler chickens. J. Agric. Dev.

[33] Liao X, Shao Y, Sun G, Yang Y, Zhang L, Guo Y, Luo X, Lu L (2020). The relationship among gut microbiota, short-chain fatty acids, and intestinal morphology of growing and healthy broilers. Poult. Sci.

[34] Elhassan M.M.O, Ali A.M, Blanch A, Kehlet A.B, Madekurozwa M.C (2019). Morphological responses of the duodenal of broiler chicks to dietary supplementation with a probiotic, acidifiers, and their combination. J. Appl. Poult. Res.

[35] Calik A, Ceylan A, Ekim B, Adabi S.G, Dilber F, Bayraktaroglu A.G, Tekinay T, Özen D, Sacakli P (2017). The effect of intra-amniotic and posthatch dietary synbiotic administration on the performance, intestinal histomorphology, cecal microbial population, and short-chain fatty acid composition of broiler chickens. Poult. Sci.

[36] Sacakli P, Çınar Ö.Ö, Ceylan A, Ramay M.S, Harijaona J.A, Bayraktaroglu A.G, Shastak Y, Calik A (2023). Performance and gut health status of broilers fed diets supplemented with two graded levels of a monoglyceride blend. Poult. Sci.

[37] Petrusewicz-Kosińska M, Przybylska-Gornowicz B, Ziółkowska N, Martyniuk K, Lewczuk B (2019). Developmental morphology of the turkey pineal organ. Immunocytochemical and ultrastructural studies. Micron.

